# Activity Patterns during Food Provisioning Are Affected by Artificial Light in Free Living Great Tits (*Parus major*)

**DOI:** 10.1371/journal.pone.0037377

**Published:** 2012-05-18

**Authors:** Mieke Titulaer, Kamiel Spoelstra, Cynthia Y. M. J. G. Lange, Marcel E. Visser

**Affiliations:** 1 Department of Animal Ecology, Netherlands Institute of Ecology, Wageningen, The Netherlands; 2 Nature Conservation and Plant Ecology Department, Wageningen University, Wageningen, The Netherlands; University of Regina, Canada

## Abstract

Artificial light may have severe ecological consequences but there is limited experimental work to assess these consequences. We carried out an experimental study on a wild population of great tits (*Parus major*) to assess the impact of light pollution on daily activity patterns during the chick provisioning period. Pairs that were provided with a small light outside their nest box did not alter the onset, cessation or duration of their working day. There was however a clear effect of artificial light on the feeding rate in the second half of the nestling period: when provided with artificial light females increased their feeding rate when the nestlings were between 9 and 16 days old. Artificial light is hypothesised to have affected the perceived photoperiod of either the parents or the offspring which in turn led to increased parental care. This may have negative fitness consequences for the parents, and light pollution may thus create an ecological trap for breeding birds.

## Introduction

Artificial light pollution is increasing worldwide and may have considerable impact on flora and fauna [1]. Despite the overwhelming amount of artificial light, especially in Europe and North America, surprisingly few studies have evaluated the effects of artificial light. What has been shown is that the impact varies with species group. Light pollution leads for instance to an increase in body mass in mice [2] and to aggregations of insects around artificial lights [1]. Birds are one of the best studied animal groups with respect to the impact of light pollution. However, the effect of artificial light has rarely been studied experimentally, which is essential to avoid the confounding effects of variables such as human disturbance which is often correlated with light pollution. One such study showed that artificial light may influence choice of nest sites in meadow birds [13].

Many studies have shown that migratory birds become disoriented and attracted to artificial light sources, disturbing migratory behaviour [e.g. 3,4,5]. Furthermore, artificial light has the potential to disrupt reproductive behaviour, as photoperiod is one of the most important cues for birds to time reproduction [6]. Female blue tits breeding close to street lights start egg laying on average 1.5 days earlier than females in dark territories [7]. In several bird species, males with territories close to artificial light sources sing earlier in the morning [7], [8]. Since onset of dawn song is an indicator that females use to assess the quality of their mate, advanced onset may disrupt adaptive mate choice and increase paternity gain of males in illuminated territories [7].

In addition to timing of seasonal events such as reproduction, artificial light also has an influence on daily activity patterns of birds such as foraging behaviour [9]. Daily activity patterns are orchestrated by internal clocks which themselves are entrained by photoperiod [10]. Photoperiod is perceived by light stimulation of photoreceptors in different brain structures that trigger behavioural and physiological responses, determining activity patterns [11], [12]. Artificial light may thus affect the perceived photoperiod which may lead to changes in daily activity patterns.

Here we experimentally study the effects of light pollution on activity patterns of free living great tits (*Parus major*) when they provision their nestlings. Great tit nestlings stay in the nest for about 18 days during which they amass ±1 gram per day. This means that parents need to provide food during most of the day, especially when the chicks get older. Survival of the fledglings is positively related to fledging mass [14], [15]. This suggests that breeding great tits could increase survival of their young either by feeding at a higher rate or for a longer period of time during the day, thereby providing more food to their nestlings and thus increasing their fledging mass.

Observational evidence suggests that birds may use night-time illumination to forage [9], [16]. We therefore hypothesized that artificial light during the nestling stage may stimulate great tits to increase the time they forage by advancing the onset of activity in the morning and/or delaying the cessation of activity in the evening, thus feeding their nestlings for a longer period of time during the day. To test this hypothesis, we used an experimental approach, to avoid the limitations of observational data. We provided great tits breeding in nest-boxes with artificial light during either the first or second half of the nestling period and measured feeding activity during the entire nestling stage, thus including the period where no light was provided.

## Results

**Figure 1:**
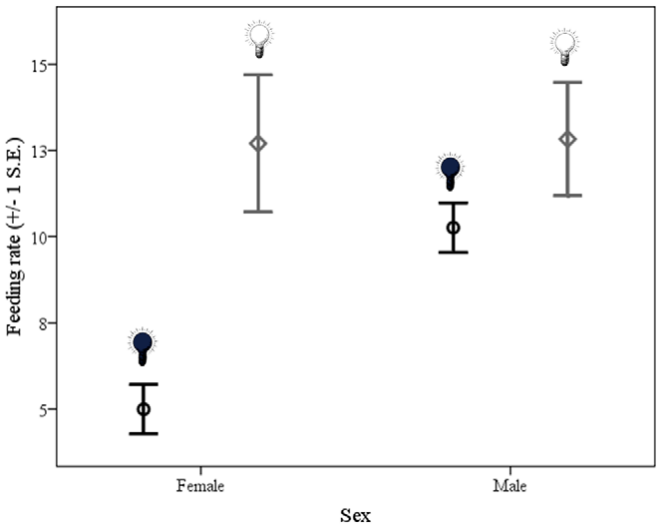
Feeding rate (number of visits to the nest per hour) for males and females in the second half of the nestling stage (nestlings of 9–16 days) per treatment (dark: black circles, light: grey diamonds).

Hatching dates varied between 2 and 11 April for first broods (N = 21) and 25 April and 3 May for second broods (N = 5) and represent a random sample of the entire population. There was no significant difference in mean hatching date between the two treatments (t = −0.79, P = 0.49). Artificial light significantly affected total activity of females in the second half of the nestling stage (P = 0.002; Table 1), but not in the first half (P = 0.71; Table 1; Treatment*Period: F_1,44_ = 5.71, P = 0.021): birds that received artificial light in the second half of the nestling stage had a higher feeding rate than birds that did not receive a light (Figure 1). There was a marginally non-significant effect for males in the same direction (F_1,18_ = 4.12, P = 0.057; Figure 1). The provisioning of artificial light did not affect the onset, cessation or duration of activity, neither as main effect (*Onset:* Females: F_1,44_ = 0.22, P = 0.64; Males: F_1,18_ = 0.19, P = 0.67; *Cessation:* Females: F_1,42_ = 0.00, P = 0.96; Males: F_1,19_ = 0.59, P = 0.45; *Duration:* Females: F_1,42_ = 0.00, P = 0.98; Males: F_1,18_ = 3.70, P = 0.07) or in interaction with any of the other tested variables (all P>0.20). Despite the higher feeding rate when light was provided in the second period, there was no difference in the increase in nestlings' mass or tarsus length between day 9 and 16 (mass: F_1,19_ = 0.06, P = 0.81; tarsus: F_1,21_ = 1.36, P = 0.26).

**Table 1:** Results of post-hoc test for the interaction between treatment and period for female feeding rate (see also Figure 1).

	estimate	s.e.	*Z*	P
Dark period 1 – dark period 2	−0.06	0.10	−0.63	0.527
Light period 1 – light period 2	0.28	0.11	2.67	0.008*
Dark period 1 – light period 1	−0.04	0.10	−0.37	0.715
Dark period 2 – light period 2	0.31	0.10	3.04	0.002*

## Discussion

A possible effect of artificial light is that it prolongs the activity period of a great tit by providing an opportunity to extend the foraging period. We did not find an effect of artificial light on the onset, cessation or duration of daily activity patterns. Our experiment shows that artificial light increases the feeding rate of females at the time the nestlings are between 9 and 16 days old.

The proportion of visits to the nest box that were logged per bird may differ for two reasons: differences in sensitivity between readers or differences in way a bird enters the nest box which may influence whether the reader detects the transponder on its leg or not. This might cause systematic differences between individuals that are not the result of the light treatment. However, readers were not changed between treatments and the reader sensitivity per nest box does not change with a change in treatment. Furthermore, we used a randomized design in which all birds received both treatments, thus cancelling out reader effects between treatments within birds.

The light we used was relatively weak; at night it was clearly visible within the nest box but it was not strong enough to illuminate a substantial part of the forest and hence it did not allow the birds to forage by it. In that sense, the period during which there was sufficient light to forage was not increased by the experimental light. This may explain why we did not find a strong effect on males, since only females stay inside the next-box during the night, thus males did not receive any light stimulation at night. Their (albeit non-significant) response may be indirect via the increased work rate of the females. There are differences in feeding rates between males and females in the control (dark) situation. An explanation for this is outside the scope of this paper but has been observed also in other Dutch populations in recent years (C.M. Lessells, pers. com.) and may thus be a specific characteristic of this population in this year.

Birds increased feeding rate in the second stage of the nestling period without increasing the length of the activity period. This is a surprising result as during the day natural daylight will overrule the experimental light. One hypothesis is that the artificial light affects the birds' perception of time of the year, i.e. days are longer later in the season and artificial light may ‘mislead’ the birds. Later fledging is related to a lower survival rate [15]. Therefore, later in the breeding season birds may work harder because they need to provide the nestlings with enough food to promote early fledging and thereby improve survival chances. However, we did not find an effect of treatment on catch-up mass (the difference in nestling mass between day 9 and 16), indicating that nestlings in boxes that received light in the second period did not grow faster than nestlings in boxes that did not receive light.

An alternative explanation for why birds would provision their brood more later in the breeding season is that the probability that a bird will breed again within the same year (starts a second brood) declines over the season [17]. Birds that perceive a long photoperiod may therefore refrain from producing a second brood and invest more in their current brood, as has been shown experimentally [18]. However, treatment did not affect the probability of starting a second brood (glm with binominal errors, correcting for laying date: χ2 = 0.26, P = 0.61) in our experiment.

Finally, it could be that the effect of light on feeding rate of the parents is through an effect on the nestlings. If light influences begging behaviour of the nestlings in such a way that nestlings in the light treatment beg more, this may induce the adults to increase their feeding rate. This would explain why not only females but also (albeit marginally non-significant) males increase their feeding rate. It could also explain why the treatment did not influence body mass because parents may bring food more often but if these prey are smaller, this may not necessarily be more food in total.

In summary, we found that artificial light increases feeding rate in female great tits in the second half of the nestling period and thus light pollution may affect breeding birds. It remains to be determined whether these effects have a positive or negative effect on the fitness of the birds. While our results indicate that the offspring may benefit from the higher provisioning rate under artificial light, this may come at the expense of parental fitness, either via reduced survival or a reduced probability to breed again in the same year. If the latter is the case, artificial light can be considered an ecological trap [19].

## Methods

### Experimental procedure

The great tits in this experiment were breeding in nest-boxes in Roekel (52°04.318′N, 5°43.084′E) a mixed woodland area in Ede, the Netherlands. This area contains 262 nest-boxes that were checked weekly during egg laying and daily close to hatching to determine the exact hatch date. Female great tits lay one egg per day and incubation lasts on average 13 days. We randomly selected 26 nest-boxes and caught the female 2 days before egg hatching to affix a metal aluminium band containing a unique ring number as well as a transponder glued to 3 colour rings. One day after hatching (day 1) we put up a box containing either an LED light (n = 13), or a similar box with no light on top of the nest-box (n = 13). The light intensity was 10 l× as measured at the level of the nest box opening. Wavelength varied between 380 and 780 nm with two peaks around 450 nm and 600 nm (See figure S1 for the complete light spectrum). We attached a transponder reader (Trovan, Dorset Group BV, Aalten, The Netherlands) on the opening of the nest-box which logged the entries and exits of the bird carrying the transponder with a unique transponder code, date and time. At day 9 the treatment was reversed. Also on day 9 both adults and chicks were caught and body mass was measured to the nearest 0.1 gram with a spring balance. Tarsus length and for adults also wing length (third primary flight feather) were measured to the nearest 0.1 mm with respectively a slide gauge and a ruler. Males and nestlings were banded with aluminium rings containing a unique number for identification and males also received a transponder. The second treatment lasted from day 9–17. At day 16 we again measured mass and tarsus length of the nestlings. Selection of nest-boxes and distribution of treatment order over the nest-boxes was performed randomly.

### Data analysis

From the reader data we calculated the onset (first activity of the day), cessation (last activity of the day), duration (difference between cessation and onset) and total activity (number of entries and exits per hour). All scores were corrected for day length and for seasonal changes in day light by calculating the scores in reference to the total period of day light at the time of measurement (calculated from sunrise to sunset). We performed four linear mixed models with onset of activity, cessation of activity, duration of activity and total activity as response variables. For males we only had data for the second treatment period, therefore separate models were run for males and females. For females, treatment (light or dark) and period (first or second half of the nestling stage) were taken as fixed factors as well as brood size, chick age and chick age^2^, April date (number of days since March 31^st^) and April date^2^, adult body condition (residuals of a regression of tarsus length and time of measurement on weight). We also tested the two-way interactions treatment*condition, treatment*period and treatment*brood size. Period*treatment nested within individual was added as a random effect for females, and treatment nested within individual for males as well as April date (as a class variable) for both males and females to control for multiple measurements taken on the same day. Final models were obtained by backward elimination of non-significant terms, starting with the highest order interactions. All analyses were performed in R 2.13.1.

### Ethics Statement

Experiments were carried out under licence NIOO 10.07 of the Animal Experimentation Committee of the Royal Dutch Academy of Sciences (DEC-KNAW).

## Supporting Information

Figure S1 **LED spectrum.**
(TIF)
