# Supplementary figures and images for: Activity Patterns during Food Provisioning Are Affected by Artificial Light in Free Living Great Tits (Parus major)
Source: PLoS One. 2012 May 18;7(5):e37377. doi: 10.1371/journal.pone.0037377 (PMC3356403; doi:10.1371/journal.pone.0037377)

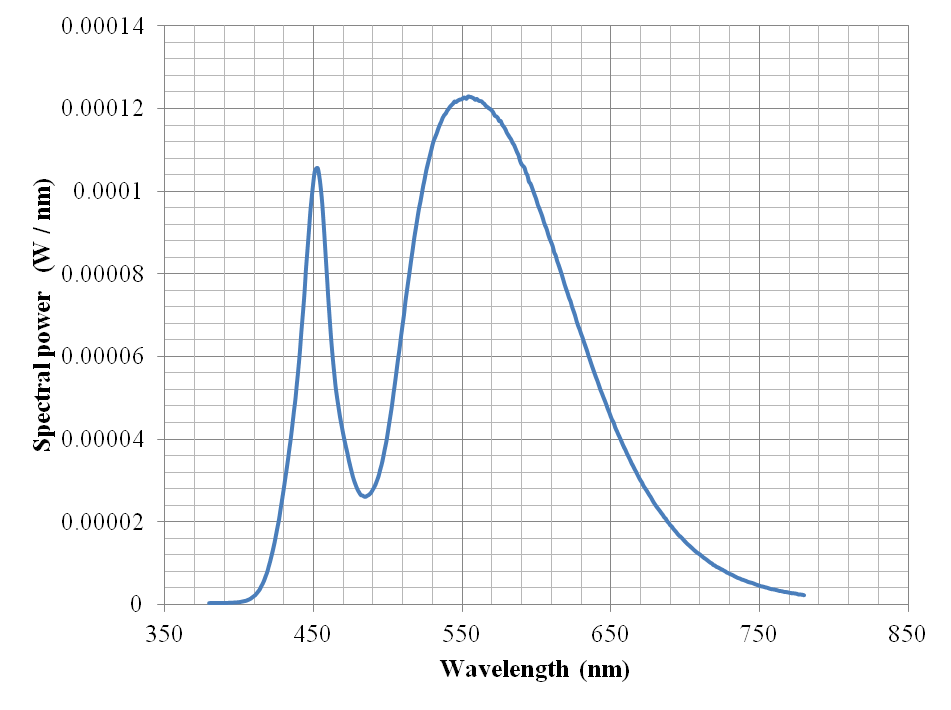

Supplement: Figure S1 — LED spectrum. (TIF) [file pone.0037377.s001.tif]
